# C3G and Ig-MPGN—treatment standard

**DOI:** 10.1093/ndt/gfad182

**Published:** 2023-08-21

**Authors:** Marina Noris, Giuseppe Remuzzi

**Affiliations:** Istituto di Ricerche Farmacologiche Mario Negri IRCCS, Clinical Research Center for Rare Diseases Aldo e Cele Daccò Ranica, Bergamo, Italy; Istituto di Ricerche Farmacologiche Mario Negri IRCCS, Clinical Research Center for Rare Diseases Aldo e Cele Daccò Ranica, Bergamo, Italy

**Keywords:** C3 glomerulopathy, complement inhibitory drugs, complement system, membranoproliferative glomerulonephritis, unsupervised cluster analysis

## Abstract

Among the broad spectrum of membranoproliferative glomerulonephritis (MPGN), immunofluorescence distinguishes C3 glomerulopathy (C3G), with predominant C3 deposits, and immunoglobulin-associated MPGN (Ig-MPGN), with combined C3 and Ig. However, there are several intersections between C3G and Ig-MPGN. Primary C3G and Ig-MPGN share the same prevalence of low serum C3 levels and of abnormalities of the alternative pathway of complement, and patients who present a bioptic pattern of Ig-MPGN at onset may show a C3G pattern in a subsequent biopsy. There is no specific therapy for primary C3G and Ig-MPGN and prognosis is unfavourable. The only recommended indications are inhibitors of the renin–angiotensin system, lipid-lowering agents and other renoprotective agents. The other drugs used currently, such as corticosteroids and mycophenolate mofetil, are often ineffective. The anti-C5 monoclonal antibody eculizumab has been tested in several patients, with mixed results. One reason for the uncertainty is the extremely variable clinical course, most likely reflecting a heterogeneous pathogenesis. An unsupervised clustering analysis that included histologic, biochemical, genetic and clinical data available at onset in patients with primary C3G and Ig-MPGN identified four clusters characterized by specific pathogenic mechanisms. This approach may facilitate accurate diagnosis and development of targeted therapies. Several trials are ongoing with drugs targeting different molecules of the complement cascade, however it is important to consider which component of the cascade may be the most appropriate for each patient. We review the current standards of treatment and discuss novel developments in the pathophysiology, diagnosis, outcome prediction and management of C3G and Ig-MPGN.

Box 1: ‘In a nutshell’.

- C3G and Ig-MPGN are complement-mediated heterogeneous glomerular diseases.
- Acquired and genetic abnormalities causing hyperactivation of the complement alternative pathway are found both in primary C3G and in primary Ig-MPGN.
- An effective treatment for primary C3G and Ig-MPGN is lacking. The long-term benefit of immunosuppression with steroids and MMF is uncertain.
- Different agents that target either the terminal pathway, or the C3 convertase of the alternative pathway or prevent C3 activation unselectively have been/are being tested in patients with primary C3G and Ig-MPGN. Preliminary results show high variability in clinical response.
- Unsupervised hierarchical clustering may facilitate the identification of groups of patients with a specific disease aetiology, who will benefit the most from distinct complement-modulating drugs.

## INTRODUCTION

The term membranoproliferative glomerulonephritis (MPGN) defines a glomerular lesion characterized by hypercellularity, mesangial matrix expansion and duplication of the glomerular basement membrane (GBM). This pattern may be seen in the setting of various secondary conditions [1, 2] as well as in primary forms that are distinguished into C3 glomerulopathy (C3G) and immunoglobulin-associated MPGN (Ig-MPGN), on the basis of immunofluorescence showing predominant or exclusive C3 deposits in C3G and combined C3 and Ig deposits in Ig-MPGN (Box 1) [1, 3]. The term C3G also includes kidney lesions that do not show the typical alterations of MPGN but share C3-dominant staining. C3G is further classified in dense deposit disease (DDD) and C3 glomerulonephritis (C3GN) based on the presence (in DDD) or absence (in C3GN) of highly electron-dense ribbon-like deposits in the GBM [1].

Primary C3G and Ig-MPGN are rare, with an estimated incidence of 1–3 cases/million/year (https://www.orpha.net/consor/cgi-bin/OC_Exp.php?lng=en&Expert=329918).

**Figure 1:**
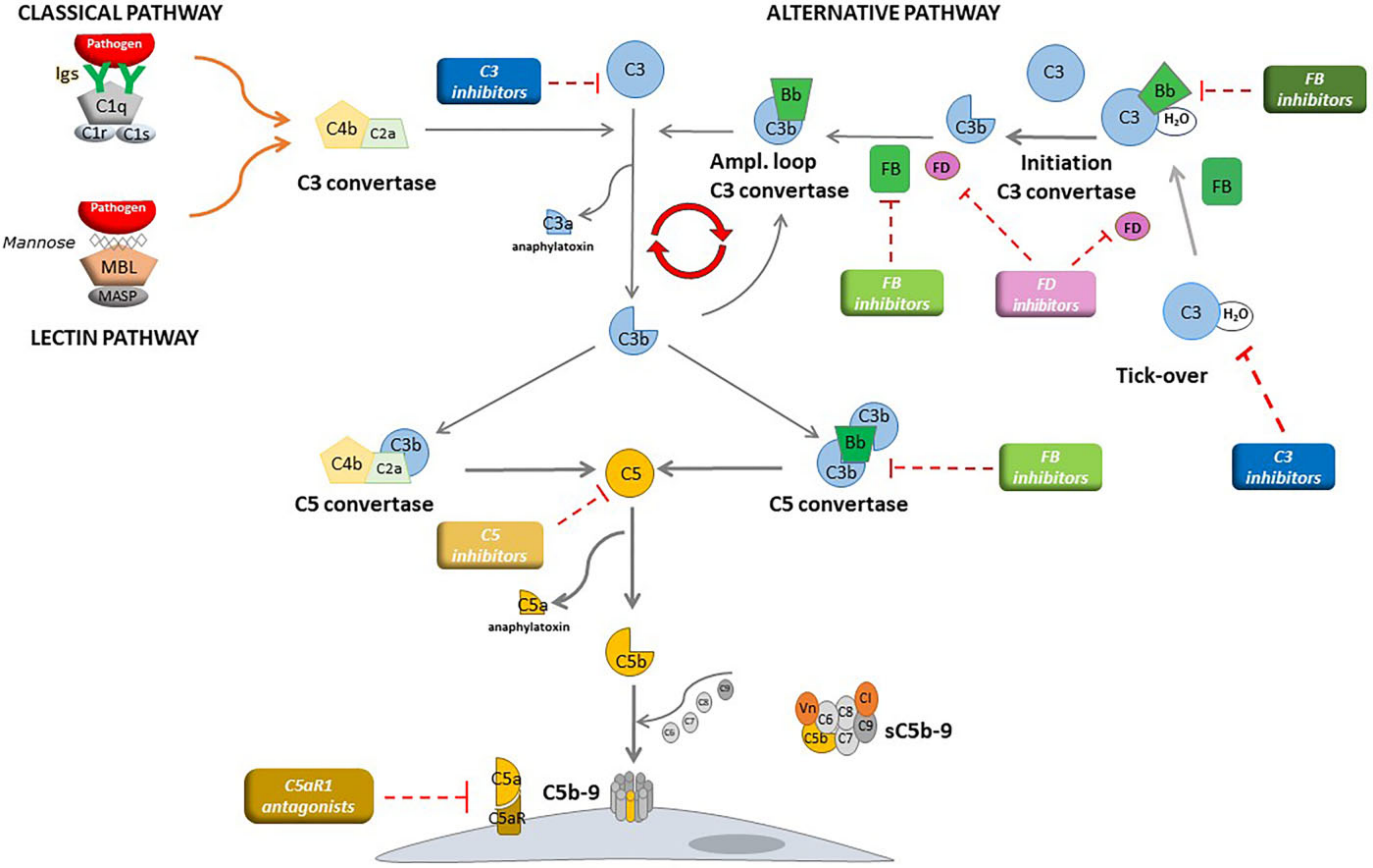
The complement cascade. The classical pathway is activated by the binding of C1q to antibody–antigen complexes, while the lectin pathway is activated by the binding of mannose-binding lectin (MBL) to mannose residues, which activates mannose-binding lectin serine peptidase (MASP) proteins. Either process results in the formation of the classical/lectin, C3 convertase complex that cleaves C3 to C3b and the anaphylatoxin C3a. The alternative pathway is continuously activated in plasma by low-grade hydrolysis of C3 (C3H2O, tick-over) that together with factor B, forms the initiation C3 pro-convertase. FD cleaves factor B to form the active alternative pathway initiation C3 convertase that cleaves C3 to C3b. Complement activation is then amplified by the covalent binding of C3b produced by all the three pathways to hydroxyl groups on cell surface carbohydrates and proteins of target cells, such as bacterial cells. This C3b binds factor B, to form the amplification loop C3 convertase C3bBb. C3b also binds to the C3 convertases, forming the C5 convertase enzymes of the classical/lectin (C4bC2bC3b) and of the alternative (C3b2Bb) pathways that lead to C5 cleavage and the formation of the anaphylatoxin C5a and of the membrane attack complex, composed of C5b, C6, C7, C8 and many copies of C9. The coloured rectangles denote the categories of complement inhibitors tested in C3G and Ig-MPGN and the red dashed lines mark their targets.

The immunofluorescence (IF)-based classification assumes that C3G arises from abnormalities in the control of the complement alternative pathway while Ig-MPGN derives from glomerular deposition of immune complexes that activate predominantly the complement classical pathway (Fig. 1). This assumption may imply that C3G and Ig-MPGN should be treated differently. However there are several intersections between primary C3G and Ig-MPGN [2]. Cohort studies have not consistently identified renal outcome differences between C3G and Ig-MPGN [3, 4] and both are characterized by a high rate of recurrence in the grafts [4]. C3G and Ig-MPGN share the same prevalence of low serum C3 levels and of alternative pathway abnormalities [5]. These include the autoantibodies C3NeFs or C5NeFs, which stabilize the C3 and C5 convertases, anti-factor B and anti-factor H antibodies, and genetic abnormalities affecting complement regulators or the two components of the alternative pathway C3 convertase, C3 and factor B (Box 1 and Fig. 1) [1, 6, 7]. Finally, there are patients who present with a bioptic pattern of Ig-MPGN and a subsequent biopsy shows isolated C3 staining compatible with a C3G [1, 6]. Taken together, these data indicate that in order to have a clear understanding of these diseases, it is necessary to go beyond the IF-based classification [8].

Here we review the current standards of treatment and discuss novel developments in the pathophysiology, diagnosis, outcome prediction and management of C3G and Ig-MPGN.

## TREATMENT STANDARDS

### Differential diagnosis

**Figure 2:**
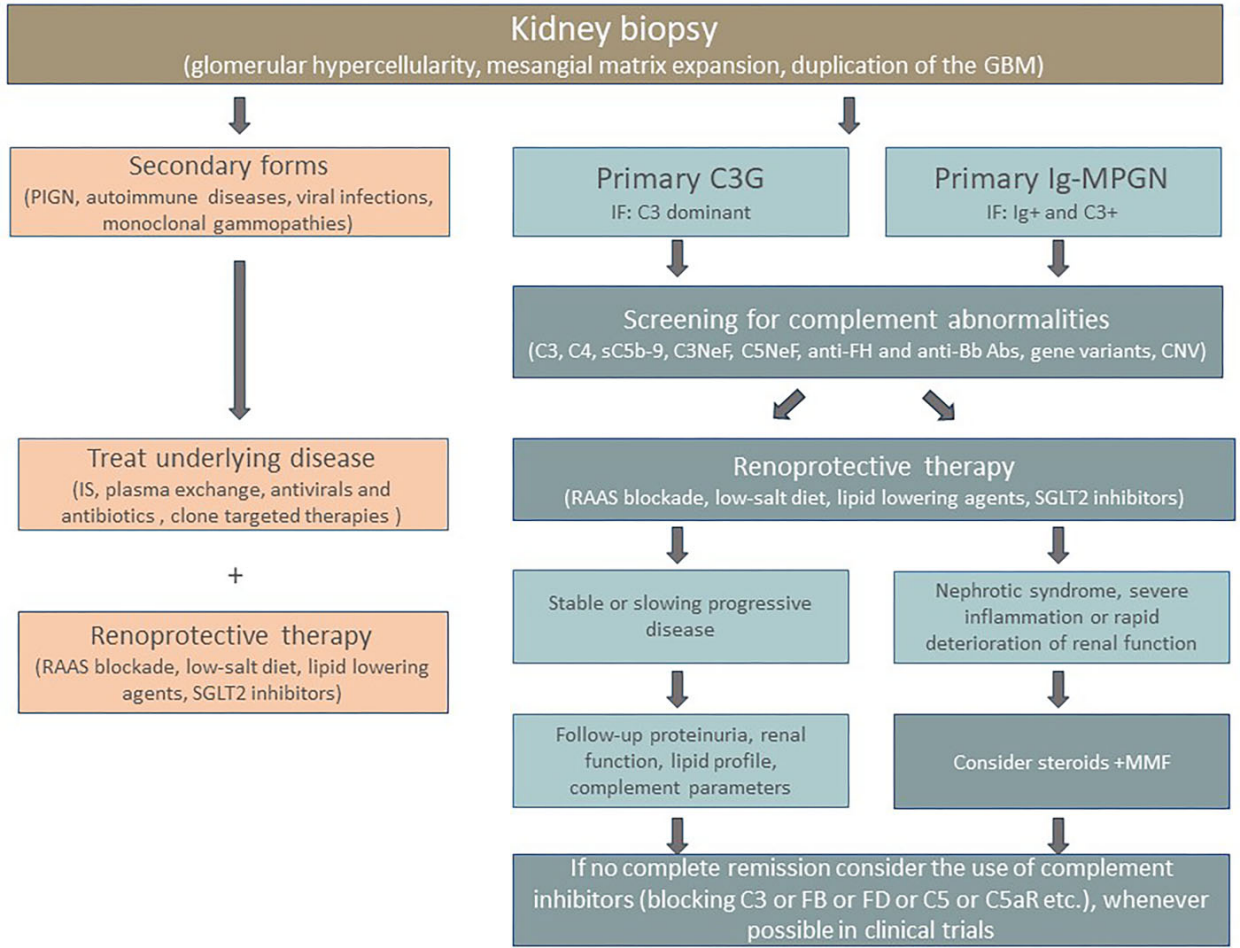
Flow chart of diagnostic algorithm and currently available treatment algorithm in C3G and Ig-MPGN. Abs, antibodies; RAAS, renin–angiotensin system; IS: immunosuppression; MMF: mycophenolate mofetil; CNV: copy number variation.

The diagnostic workup should identify secondary forms that require specific treatments targeted to the underlying conditions (Fig. 2).

For C3G, differential diagnosis includes post-infectious glomerulonephritis (PIGN), an acute form of nephritis linked to transient complement alternative pathway activation triggered by the immune reaction to bacterial infections. PIGN occurs mainly in children, but rarely it is also observed in elderly adults, and histologically presents with endocapillary and exudative proliferative glomerulonephritis and a bright glomerular C3 staining often without Ig [9]. At electron microscopy the most common finding in PIGN is subepithelial hump-shaped deposits. PIGN is self-limited and reversible with resolution of infection in the vast majority of cases. However, glomerular lesions in PIGN and C3G often overlap and in a subgroup of patients PIGN progresses to chronic C3G with C3 consumption and persistent proteinuria [1].

The Ig-MPGN pattern may occur in the context of autoimmune diseases (lupus nephritis, Sjögren’s syndrome, cryoglobulinemia, mixed connective tissue disease), chronic viral (HBV, HCV, HIV) and bacterial (endocarditis, chronic abscess) infections, and sickle cell anaemia [2]. In these secondary cases, treatment of the underlying condition—immunosuppression for autoimmune diseases, antiviral drugs and antibiotics for viral and bacterial infections—often also improves kidney injury and dysfunction (Fig. 2) [2, 10, 11].

Both C3G and Ig-MPGN may occur in the context of monoclonal gammopathies, characterized by the proliferation of a single clone of Ig-producing lymphocytes or plasma cells. The clinical spectrum of monoclonal gammopathies includes monoclonal gammopathy of undetermined significance, Waldenström macroglobulinemia, lymphoproliferative disorders and multiple myeloma [12–14]. Typical immunofluorescence features are subendothelial deposits of monoclonal Ig, most commonly IgG with κ and λ restriction [9]. Treatment of the haematologic malignancy with clone-targeted therapies (with a bortezomib-based regimen and more recently with the human monoclonal anti-CD38 antibody daratumumab) was associated with improvement of clinical manifestations and glomerular lesions, and showed a therapeutic superiority versus conventional immunosuppression. [9, 12]

### Renoprotective strategies

A universally effective treatment for primary C3G and Ig-MPGN is lacking, and there is an open debate about how to treat patients (Box 1). One reason for this uncertainty is the extremely variable clinical course, which ranges from chronic or intermittent haematuria and/or proteinuria associated with a rather stable renal function, to subacute/chronic progressive deterioration of renal function. A few patients even manifest an acute glomerulonephritis rapidly progressing to end-stage renal disease (ESRD). The relative frequency of the different clinical forms is not known. Another bias is due to the fact that several studies assessing treatments were enriched in patients with rather aggressive disease, or included patients with both primary and secondary Ig-MPGN [1, 6].

The only recommended indication for the management of patients with primary C3G and Ig-MPGN, proteinuria and preserved renal function is an antiproteinuric therapy with inhibitors of the renin–angiotensin system, which has been shown to effectively slow renal disease progression in other glomerular diseases [6, 15, 16]. Additional kidney protective measures include a low-salt diet and treatment of dyslipidaemia (Fig. 2, Table 1) [17]. More recently, inhibitors of the sodium-glucose cotransporter 2 (SGLT2) have been shown to slow renal disease progression in diabetic but also in non-diabetic proteinuric nephropathies [18, 19].

**Table 1:** Drugs currently used in primary forms of C3G and Ig-MPGN^a^.

Drugs	Dose	Indication	Markers of response	Precautions and toxicities
ACEi and/or angiotensin type 1 receptor antagonists	Maximal tolerated dose	All patients, to control proteinuria and blood pressure	↓ proteinuria, normalization of blood pressure (120/80)	Dry cough (ACEi), AKI especially in children, angioedema, hyperkalemia, light-headedness and dizziness in patients with hypotension
Lipid-lowering agents (statins, lifestyle change for triglycerides)	According to specific drug labels	Mandatory in patients with nephrotic syndrome	Normalization of total cholesterol, of LDL and of triglyceride levels	Monitor CPK (statins). Muscle pain and damage, liver damage, increased blood glucose
Dietary sodium restriction	Sodium consumption <2 g/day (<5 g/day of salt)	Mandatory in patients with nephrotic syndrome	↓ oedema, ↓ body weight, ↓ blood pressure	Low compliance
SGLT2 inhibitors	According to specific drug labels	Adult patients with renal impairment and/or proteinuria	GFR improvement/stabilization, normalization of glucose levels, ↓ proteinuria	Mycotic genital infections. Not approved for children
Prednisone	Oral, 1–2 mg/kg/day, for 4 weeks, then taper and discontinue	Severe proteinuria, rapid progressive disease, severe inflammation in biopsy	↓ proteinuria (complete: <0.3/24 h; or partial: >0.3 g; but <3.5 g/24 h, remission), GFR stabilization	Obesity, diabetes, hypertension, osteopenia, impaired growth. Avoid prolonged exposure. Calcium and vitamin D supplement
MMF	600 mg/m^2^/day in 2 doses initially then titrate individually according to tolerance	Severe proteinuria, rapid progressive disease, severe inflammation in biopsy	↓ proteinuria (complete or partial remission), GFR stabilization	May cause abdominal pain, loss of appetite, anaemia and leukopenia. Check CBC and liver enzymes. Must not be taken by pregnant women
Eculizumab	Adults: 900 mg i.v. qWeek for 4 weeks followed by 1200 mg every other week. Children: dose adjusted for weight (kg)	Rapidly progressive disease, severe glomerular inflammatory lesions, overlap with aHUS	↓ proteinuria, normalization of plasma sC5b-9 levels, GFR stabilization	Vaccinations and antibiotic prophylaxis to prevent infections by *Neisseria* and other encapsulated bacteria. Not approved for C3G/Ig-MPGN

^a^KDIGO 2021 Clinical practice guideline for the management of glomerular diseases, supplement to *Kidney International* 2021;11:4S; and World Health Organization (WHO). Available at: https://www.who.int/news-room/fact-sheets/detail/salt-reduction.

ACEi, angiotensin-converting enzyme inhibitors; CPK, creatine phosphokinase; CBC, complete blood cell count; AKI, acute kidney injury; LDL, low-density lipoprotein.

### Immunosuppression

For patients with nephrotic syndrome or progressive deterioration of renal function despite renoprotective therapy, non-specific immunosuppression is often prescribed to target the inflammatory component in the glomerular microenvironment and decrease the production of complement activating autoantibodies (Table 1, Fig. 2). Current guidelines suggest attempting a trial with oral prednisone and mycophenolate mofetil (MMF) in adults and children [1, 20, 21], on the basis of retrospective multicentre studies in patients with C3G that have shown a higher rate of remission and lower risk of ESRD versus patients who received other immunosuppressives or conservative management alone (Table 2) [22, 23]. The therapeutic effect of steroids plus MMF was observed in patients with autoantibody-mediated (C3NeFs) disease and to lesser extent also in those with genetic causes. However, the response was not consistent, with only 36% of patients achieving a complete remission [22]. There is still uncertainty about the optimal duration of treatment, and about whether clinical improvement will translate to long-term benefits (Box 1 and Box 2). Actually, 30%–50% of patients relapsed when treatment was stopped [22], which indicates that MMF and steroids do not target the primary pathogenic mechanisms underlying C3G.

**Table 2:** Published clinical studies focused on MMF in C3G and primary Ig-MPGN.

	Diagnosis and treatment groups		Response vs pre-treatment		
References	C3G	Ig-MPGN	Proteinuria remission (CR or PR)	Renal function	Follow-up comparison with controls
Jones *et al*. (S1)		MMF+S (*n* = 5)	Yes (mean)	= (mean)	MMF + S vs no IS: proteinuria ns, serum creatinine ns.
		No IS (*n* = 6)	No (mean)	↓ (mean)	
Yuan *et al*. (S2)		MMF+S (*n* = 13)	Yes (mean)	↑(mean)	No comparison
Haffner *et al*. (S3)	MMF + S + P (*n* = 4)		Yes (*n* = 3)	↑ (*n* = 3)	No comparison
			No (*n* = 1)	= (*n* = 1)	
Caliskan *et al*. (S4)	MMF (*n* = 27)			GFR ↓ ≥50%: (*n* = 8/27)	No difference vs S or CP and No IS
	S or CP (*n* = 23)			GFR ↓ ≥50%: (*n* = 4/23)	
	No IS (*n* = 16)			GFR ↓ ≥50%: (*n* = 5/16)	
Bharati *et al*. (S5)	MMF ± S (*n* = 17)		Yes (*n* = 11)	↑ (*n* = 4), = (*n* = 7)	No comparison
			No (*n* = 6)	↓ (*n* = 6)	
Avasare *et al*. (S6)	MMF + S (*n* = 30)		Yes (*n* = 20), no (*n* = 10)	= (yes), ↓ (no)	No statistical comparison
	S ± CNI ± R ± CP (*n* = 43)		Yes (*n* = 15/43)	NA	
Yeter *et al*. (S7)	MMF + S (*n* = 7)		Yes (*n* = 3/7)	= (*n* = 5), ↓ (*n* = 2)	No difference vs S + CP
	S + CP (*n* = 14)		Yes (*n* = 8/14)	= (*n* = 6), ↓ (*n* = 8)	
Rabasco *et al*. [23]	MMF + IS (*n* = 22)		Yes (*n* = 19/22)	=/↑ (*n* = 22), RF (0)	Remission: MMF + IS better vs the other groups
	Other IS (*n* = 18)		Yes (*n* = 9/18)	=/↑ (*n* = 8), RF (*n* = 3)	
	No IS (*n* = 20)		Yes (*n* = 5/20)	=/↑ (*n* = 6), RF (*n* = 7)	
Caravaca-Fontan *et al*. (S8)	MMF + S (*n* = 42)		Yes (*n* = 33/42)	=/↑ (*n* = 33), RF (*n* = 6)	Kidney survival: MMF + S better vs the other groups
	Other IS (*n* = 29)		Yes (*n* = 7/29)	=/↑ (*n* = 7), RF (*n* = 17)	
	Ecu (*n* = 9)		Yes (*n* = 3/9)	=/↑ (*n* = 3), RF (*n* = 6)	
	No IS (*n* = 17)		Yes (*n* = 3/17)	=/↑ (*n* = 3), RF (*n* = 11)	
Ravindran *et al*. (S9)	MMF ± S (*n* = 24)		Yes (*n* = 3/24)	RF (*n* = 9)	No difference vs S and No IS
	S (*n* = 12)		Yes (*n* = 5/12)	RF (0)	
	No IS (*n* = 34)		Yes (*n* = 13/34)	RF (*n* = 3)	
Khandelwal *et al*. [26]	MMF ± IS (*n* = 40)	MMF + IS (*n* = 11)	Yes (*n* = 14/51)	NA	No significant impact of MMF on outcome
	Other IS (*n* = 26)	Other IS (*n* = 7)	Yes (*n* = 12/33)	NA	
Pinarbasi *et al*. (S10)	MMF/AZA (*n* = 31)		Yes (*n* = 13/31)	NA	No difference vs S and Other IS
	S (*n* = 11)		Yes (*n* = 9/11)	NA	
	Other IS (*n* = 16)		Yes (*n* = 7/16)	NA	

S, corticosteroids; P, plasma; CP, cyclophosphamide; IS, immunosuppressants; CNI, calcineurin inhibitors; R, rituximab; Ecu, eculizumab; AZA, azathioprine; CR, complete remission; PR, partial remission; RF, renal failure; =, unchanged; ↑, improved; ↓, worsened; NA, not available; ns, not significant.

The benefit of immunosuppression in primary Ig-MPGN is even more uncertain. Calcineurin inhibitors, cyclophosphamide, oral and intravenous corticosteroids, and MMF have been attempted, but the data on outcome are scanty and controversial. While in a few studies a protracted course of oral prednisone in children with primary Ig-MPGN showed some benefit in slowing disease progression [24], there is no evidence that steroids are effective in adults [1, 24]. Of relevance, two recent retrospective studies in a multicentre [25] and a single-centre paediatric cohort [26] which included patients with both C3G and primary Ig-MPGN did not identify an association between immunosuppression treatment (mostly corticosteroids and MMF) and improvement in renal disease outcome or in histology (Table 2).

The anti-CD20 antibody rituximab which specifically depletes B lymphocytes has been proven effective in patients with secondary forms and monoclonal immune deposits. This treatment was also attempted in a few patients with primary C3G or Ig-MPGN to target C3NeF-producing cells, but published results about the effect on kidney abnormalities are essentially negative [27].

### Complement C5 inhibition

The anti-C5 monoclonal antibody eculizumab [28] (Figs 1 and 2) has been tested in single C3G or Ig-MPGN patients, small retrospective series [29, 30] and in an off-on-off-on study, the EAGLE (Evaluating the Morphofunctional Effects of Eculizumab Therapy in Primary Membranoproliferative Glomerulonephritis) trial [31], with mixed results (Table 3). In some patients, proteinuria improved and renal function stabilized; however, the response to treatment was mostly partial in the long-term and about half patients did not benefit from the treatment. Published data suggested that elevated levels of the soluble form of the terminal complement complex (sC5b-9) could be a marker of eculizumab responsiveness [32]. However, the EAGLE trial which recruited 10 patients with very high levels of sC5b-9 documented a consistent decrease in proteinuria only in three of them, despite sC5b-9 being fully normalized by the drug in all patients [31] (Table 3).

**Table 3:** Outcome of published patients with C3G or primary Ig-MPGN treated with eculizumab.

	Diagnosis			Response		
References	C3G (No.)	Ig-MPGN (No.)	Plasma sC5b-9	Proteinuria reduction	Renal function	Histology
Vivarelli *et al*. (S11)	1 native		Elevated	Yes	= (normal)	Improved
Daina *et al*. (S12)	1 native		Elevated	Yes	Improved	ND
Radhakrishnan *et al*. (S13)		1 native	Elevated	Yes	Improved	ND
Bomback *et al*. (S14), Herlitz *et al*. (S15)	3 native + 3 tx		Elevated (*n* = 2)	= (*n* = 1 normal), no (*n* = 1)	Improved (*n* = 1), = (*n* = 1)	Improved (*n* = 2)
			Normal (*n* = 3)	= (*n* = 1 normal), no (*n* = 2)	Improved (*n* = 1), worsened (*n* = 2)	=, worsened, ND
			ND (1)	Yes (*n* = 1)	=	Improved
McCaughan *et al*. (S16)	1 tx		ND	Yes	Improved	=
Levart *et al*. (S17)	1 native		Elevated	Yes	=	Improved
Kerns *et al*. (S18)		1 native	Elevated	Yes	=	ND
Gurkan *et al*. (S19)	1 tx		Elevated	Yes initially, then no	=	Worsened
Payette *et al*. (S20)	1 native		Elevated	Yes	= (normal)	Improved
Rousset-Rouviere *et al*. (S21)	1 native		ND	Yes	Improved	ND
Oosterveld *et al*. (S22)	5 native		Elevated (*n* = 3)	Yes (*n* = 3)	Improved (*n* = 3)	=(*n* = 1), ND (*n* = 2)
			Normal (*n* = 2)	Yes (*n* = 2)	Improved (*n* = 2)	ND (*n* = 2)
Inman *et al*. (S23)	1 native		Elevated	Yes	Improved	ND
Tran *et al*. (S24)	1 native		Elevated	Yes	Improved	ND
Le Quintrec *et al*. (S25)	2 native + 1 tx		Elevated (*n* = 2)	Yes (*n* = 2)	Yes (*n* = 2)	Improved (*n* = 2)
			Normal (*n* = 1)	Yes (*n* = 1)	Yes (*n* = 1)	Improved (*n* = 1)
Lebreton *et al*. (S26)	2 native + 1 tx	1 native	Elevated (*n* = 2)	Yes (*n* = 2)	= (normal, *n* = 2)	= (*n* = 2)
			Normal (*n* = 2)	Yes (*n* = 1), no (*n* = 1 tx)	= (*n* = 1), ND (*n* = 1 tx)	ND (*n* = 2)
Welte *et al*. (S27)	4 native + 3 tx		Elevated (*n* = 2)	Yes (*n* = 2)	Improved (*n* = 1), = (*n* = 1)	ND (*n* = 2)
			ND (*n* = 5)	Yes, no, yes, no, no	=, =, improved, worsened, worsened	ND (*n* = 5)
Garg *et al*. (S28)	1 tx		ND	Yes	Improved	Improved
Ozkaya *et al*. (S29)	1 native		ND	Yes	= (normal)	ND
Sanchez-Moreno *et al*. (S30)	1 tx		ND	Yes	Improved	Improved
Besbas *et al*. (S31)	1 native		ND	No	= (normal)	ND
Ruggenenti *et al*. [31], Carrara *et al*. (S32)	4 native	6 native	Elevated (*n* = 10)	Yes (*n* = 4 Ig-MPGN)	= (*n* = 4)	Improved (*n* = 1)
				No (*n* = 6)	= (*n* = 5), ESRD (*n* = 1)	Worsened (*n* = 1)
						ND (*n* = 8)
Le Quintrec *et al*. [32].	26 native		Elevated (*n* = 16)	Yes (*n* = 9), no (*n* = 7)	= (*n* = 9), worsened (*n* = 7)	Improved (*n* = 9),= (*n* = 2)
			Normal (*n* = 4),	Yes (*n* = 1), no (*n* = 3)	= (*n* = 1), worsened (*n* = 3)	ND (*n* = 15)
			ND (*n* = 6)	Yes (*n* = 2), no (*n* = 4)	= (*n* = 2), worsened (*n* = 4)	
Spartà *et al*.(S33)	1 native	1 native	Elevated (*n* = 2)	Yes (*n* = 2)	= (*n* = 2)	ND
Chanchlani *et al*. (S34)		1 native	Elevated	Yes	Improved	ND
Holle *et al*. (S35)	2 native + 1 tx		Elevated (*n* = 2)	Yes (*n* = 2)	Improved, (*n* = 2)	ND (*n* = 2)
			ND (*n* = 1)	Yes (*n* = 1)	= (*n* = 1)	ND (*n* = 1)
Sahin *et al*. (S36)	1 tx		ND	Yes	Improved	ND
Kojc *et al*. (S37)	2 native	1 native	Elevated (*n* = 3)	Yes (*n* = 2),	Worsened (*n* = 1) = (normal *n* = 1)	Worsened, ND
				No (*n* = 1)	= (normal)	= (*n* = 1)
Regunathan-Shenk *et al*. (S38)	5 tx		Elevated (*n* = 1)	No (*n* = 1)	Worsened (*n* = 1)	ND (*n* = 1)
			Normal (*n* = 2)	Yes (*n* = 1), no (*n* = 1)	= (*n* = 1), worsened (*n* = 1)	ND (*n* = 2)
			ND (*n* = 2)	Yes (*n* = 1), no (*n* = 1)	Worsened (*n* = 2)	ND (*n* = 2)
Yeter *et al*. (S7)	1 native + 2 tx		ND (*n* = 3)	Yes (*n* = 1), no (*n* = 2)	= (*n* = 1), improved (*n* = 1), worsened (*n* = 1)	ND (*n* = 3)
Yazilitas *et al*. (S39)	1 native		ND	Yes	Improved	ND
Pinarbasi *et al*. (S10)	18 native		ND (*n* = 18)	ND (*n* = 18)	Improved (*n* = 4) = (normal *n* = 8), worsened (*n* = 6)	ND (*n* = 18)
Kasahara *et al*. (S40)	1 native		Elevated	Yes	Improved	Improved
Blatt *et al*. (S41)	1 native		ND	Yes	Improved	Improved
Naseer *et al*. (S42)	1 tx		ND	Yes	=	ND
.Riedl Khursigara *et al*. [35]	1 native		Elevated	Yes	Improved	Worsened
Balestra *et al*. (S43)	1 native		Elevated	No	=	ND

=, unchanged; ND, not done; tx, post-transplant.

**Figure 3:**
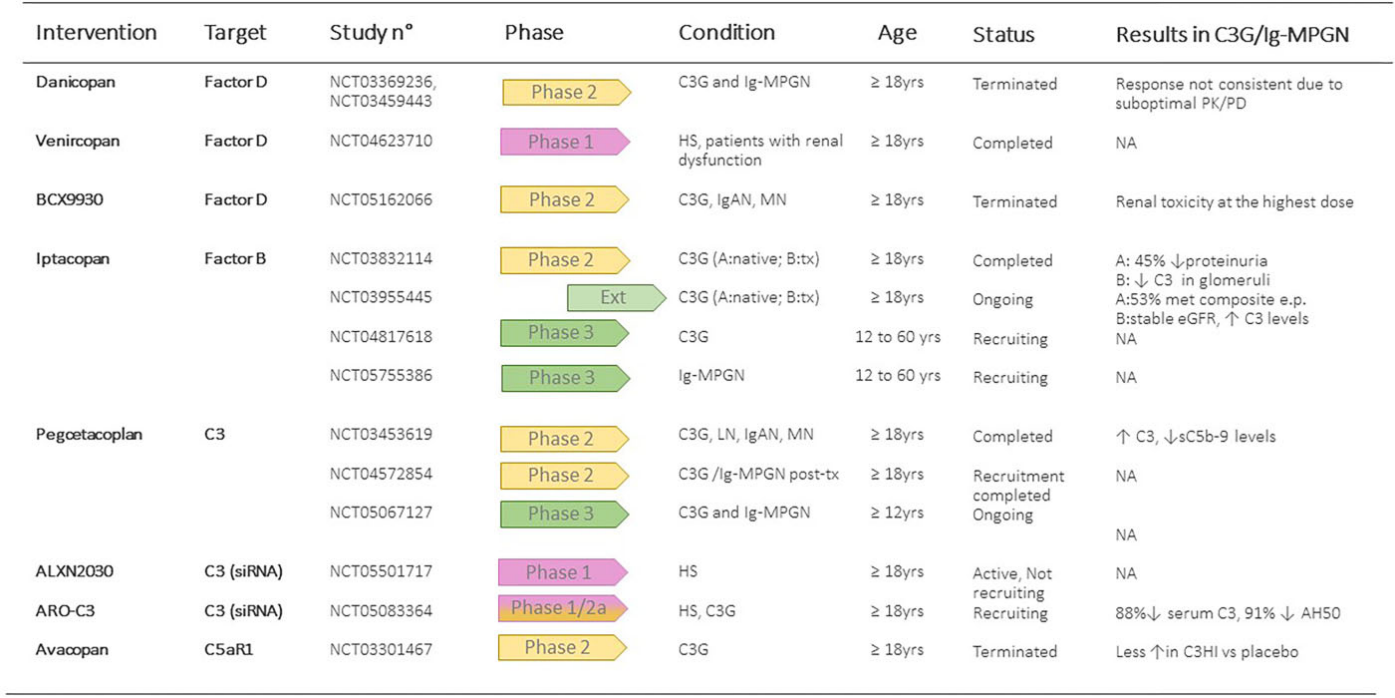
Interventional clinical trials targeting complement in C3G and Ig-MPGN. siRNA, small interfering RNA; HS, healthy subjects; IgAN, IgA nephropathy, MN, membranous nephropathy; LN, lupus nephritis; tx, post-transplant recurrence; PK, pharmacokinetics; PD, pharmacodynamics; NA, not available; e.p.: endpoint; C3HI: C3G histology index of chronicity score. All quoted trials are registered at https://www.clinicaltrials.gov/. Detailed information about participation criteria, contacts and location are provided in the website.

Interestingly, in a retrospective French study with 26 patients, the subgroup with the better clinical response to C5 inhibiton had rapidly progressive disease and more intense glomerular inflammatory lesions before treatment [32] (Table 3 and Box 2). This finding suggests that response to eculizumab may depend on preventing the formation of the potent anaphylatoxin C5a (Fig. 1), which limited leukocyte infiltration and protected the kidney from complement-mediated inflammation, rather than via reduction of C5b-9 deposition in the glomeruli. In this view, inhibitors of C5a/C5aR signalling could be as effective in C3G/Ig-MPGN as C5 inhibitors. Along this line, preliminary results of a phase 2 trial in C3G patients treated with avacopan, an oral drug that blocks the interaction of C5a with its receptor C5aR1 (NCT03301467), showed a partial effect in attenuating disease progression as compared with placebo [33]. Indeed the increase in C3G histology index of chronicity score at Week 26 was lower with avacopan compared with placebo (Fig. 3) [33].

Altogether, published results are insufficient to recommend C5 inhibition as treatment of primary C3G and Ig-MPGN, particularly for patients with subacute/chronic lesions which appear to be less prone to respond. There is no clear marker that predicts response to C5 inhibition. C3GN, DDD and Ig-MPGN patients did not show remarkable differences in this regard. C5 inhibition may be attempted in cases with acute crescentic lesion, as well as in patients with mixed thrombotic microangiopathy (TMA)/C3G lesions in whom C5-dependent glomerular endothelial injury play a pathogenic role as in atypical haemolytic uremic syndrome (aHUS) (Table 1 and Fig. 2) [34]. Patients with severe proteinuria may suffer from accelerated loss of drug in the urine, and may benefit from close monitoring and individualized therapy. This was the case for a boy with C3GN and the nephrotic syndrome in whom pharmacokinetic studies revealed low levels of eculizumab and MMF, and intensification of both treatments improved outcome [35].

The experience with C5 inhibitors has taught us that activation of the terminal complement pathway is a secondary event in C3G and Ig-MPGN that in some patients may exacerbate inflammation and kidney injury. Upstream C3-dependent pathways that cannot be blocked by C5 inhibitors guide the primary events that cause the accumulation of C3 activation fragments within immune deposits and the associated glomerular lesions.

### Post-transplant recurrence

Recurrence of C3G or Ig-MPGN in the kidney allograft represents an important cause of graft loss and morbidity [36] and at present no effective treatment is available to prevent or treat recurrences. A retrospective analysis of a multicentre cohort of kidney transplant patients included 34 with C3G and 132 with primary Ig-MPGN of whom 41 had recurrent disease in the graft, despite the fact that the large majority were receiving calcineurin inhibitors, steroids and MMF as maintenance anti-rejection therapy. Intensified immunosuppression with corticosteroids and MMF dose increase, and additional strategies to remove autoantibodies (rituximab and plasma exchange) were rather ineffective. Indeed, only 5%–6% of recurrent patients achieved complete remission, and 25/41 experienced kidney graft failure during follow-up [36]. Results of prophylaxis with rituximab or anti-C5 therapy administered pre-transplant have been variable, likely reflecting the heterogeneity of disease mechanisms [37].

## NEW DEVELOPMENTS

Despite the IF-based classification providing some advances toward an aetiology-based diagnosis, the heterogeneity of C3G and Ig-MPGN makes it challenging to predict what the effect of the disease over time is and what can be done to prevent it.

To address this hurdle we used a mathematical approach termed ‘unsupervised cluster analysis’ [38] in a cohort of 173 C3G and Ig-MPGN patients [5]. This approach, which places patients with many commonalities close together, so that differences within a cluster are small, whereas differences between clusters are large, has been previously applied to identify disease subtypes of Parkinson’s, Alzheimer’s, asthma and other conditions [38, 39]. We included in the analysis histologic, biochemical, genetic and clinical parameters that were available at disease onset, and identified four clusters [5]. Clusters 1–2 included patients with complement alternative pathway activation at both the C3 and C5 levels in the circulation, and were distinguished by markers of activation of the classical pathway in the biopsy in Cluster 2. In Cluster 3, fluid-phase alternative pathway activation at the C3 level predominated, and most patients had highly electron-dense deposits in the GBM. Finally, Cluster 4 showed solid phase-restricted complement activation with glomerular C3 deposits and a normal complement profile in the blood. This cluster showed the lowest prevalence of genetic and acquired complement abnormalities.

Clusters did better at predicting renal survival than the classification into DDD, C3GN and Ig-MPGN, with Cluster 4 showing the highest risk of ESRD during follow-up [5]. Notably, while the large majority of DDD patients fell into Cluster 3, C3GN and Ig-MPGN patients were distributed among clusters, reinforcing the overlap between C3GN and IC-MPGN and the heterogeneity of the these histologic groups [5].

The cluster approach, which was subsequently validated by an independent group in another cohort of C3G and Ig-MPGN patients [40], might hopefully help clinicians to identify group of patients who will benefit the most from distinct complement-modulating drugs (Box 1).

This aspect is particularly topical, since different agents that either specifically target the alternative pathway or prevent C3 activation unselectively via the three activation pathways are being tested in patients with C3G and Ig-MPGN (Figs 1 and 3 and Box 1) [41].

Among alternative pathway inhibitors, danicopan [42] is a first-generation orally active inhibitor of factor D (FD), the serine protease that cleaves factor B in the proconvertase C3bB, and converts it into the active C3 convertase enzyme of the alternative pathway, C3bBb (Fig. 1). *In vitro*, the compound inhibited complement-mediated haemolysis of erythrocytes from patients with paroxysmal nocturnal haemoglobinuria and effectively inhibited haemolysis *in vivo* in 12 patients with an inadequate response to eculizumab [42].

Two phase 2 studies (NCT03369236, NCT03459443) have recently evaluated the pharmacokinetics/pharmacodynamics, the efficacy and safety of danicopan in patients with C3G and in patients with C3G or Ig-MPGN, respectively [43, 44].

Danicopan reduced alternative pathway activity shortly after administration. However, the activity recovered within a few hours. Neither C3 nor sC5b-9 levels reached their normal ranges in either study, a finding that confirms that danicopan plasma concentrations were largely suboptimal for sustained inhibition of alternative pathway activity. Consequently, limited clinical response was observed in the two studies: biopsy composite index and activity index scores marginally improved from baseline in danicopan-treated patients, but mean chronicity indexes did not improve or deteriorated and glomerular C3 staining did not change from baseline [43, 44] (Fig. 3).

FD is a highly active enzyme, indeed *in vitro* and *in vivo* studies indicated that >90% of FD inhibition is required to block efficiently the alternative pathway [45]. C3G and Ig-MPGN patients may need an higher degree of FD inhibition to prevent even minute formation of a dysregulated C3 convertase that would maintain hyperactivity of the alternative pathway, C3b deposition in glomeruli and disease progression. In the two danicopan studies baseline FD concentrations were inversely correlated with estimated glomerular filtration rate (eGFR) [43, 44]. This finding is consistent with the fact that FD is filtered through the glomerulus and is catabolized in the proximal tubule [46], so that patients with renal impairment have higher than normal FD levels. In addition, the degree of alternative pathway inhibition and clinical response to FD inhibition showed large inter-individual variations [43, 44].

A second-generation, more potent FD inhibitor (venircopan) is under investigation in a phase 1 study (NCT04623710, Fig. 3), and in a phase 2 trial in patients with lupus nephritis or IgA nephropathy (NCT05097989). In preliminary studies, another oral FD inhibitor (BCX9930) demonstrated >98% suppression of the alternative pathway for 24 h post-dosing in C3G patients. These results supported a phase 2 study of BCX9930 [47] administered for 24 weeks to adults with either C3G, IgA nephropathy or membranous nephropathy [NCT05162066, RENEW (BCX9930 for the Treatment of C3G, IgAN, and PMN), Fig. 3]. However, evidence of renal toxicity, most likely due to crystal formation in the kidneys in some patients, led to revised protocols at a reduced dose, and finally the company discontinued development of this drug [47].

Failure of trials with FD inhibitors have provided the important lesson that it is crucial to invest in specific *ex vivo* tests of complement activity—such as the haemolytic assay, C3 convertase activity and stability assays [7, 48], total complement activity tests and plasma levels of complement activation products—to evaluate the ideal degree of complement inhibition in each patient and to identify individualized dosing and treatment.

More promising data with alternative pathway inhibition come from iptacopan (LNP023), a small, orally active molecule that binds to factor B and Bb. Iptacopan does not prevent the formation of the C3 convertase, but it specifically inhibits C3 convertase enzymatic activity, blocking the cleavage of C3 and the activation of the amplification loop, without affecting the classical/lectin pathways [48]. In preclinical studies, iptacopan blocked complement activation in sera from C3G patients and inhibited the activity of the C3 convertase stabilized by C3NeFs [48].

In an open-label phase 2 study (NCT03832114), 12-week treatment with iptacopan was associated with a significant (45%) reduction in proteinuria in patients with C3G in the native kidney (cohort A), and with a significant reduction of C3 deposits in the kidney grafts of patients with recurrent C3G post-transplant (cohort B). The treatment was well tolerated, with no treatment-emergent severe adverse events (Fig. 3). Preliminary results at 12 months from 26 patients who entered the long-term extension study (NCT03955445) showed that 53% of patients from Cohort A met the composite endpoint of stable/improved eGFR, ≥50% reduction from baseline in urine protein/creatinine ratio (uPCR) and ≥50% increase in serum C3. In patients from Cohort B, eGFR was stable and C3 levels increased by 96% [49]. A multicentre, double-blind, placebo-controlled phase 3 study (APPEAR-C3, NCT04817618, Fig. 3) [50] is ongoing in adolescent and adult (12–60 years) C3G patients who are randomized to either iptacopan or placebo for 6 months, followed by open-label treatment with iptacopan for 6 months. The primary objective is to evaluate the efficacy of iptacopan versus placebo on proteinuria reduction. Secondary endpoints will include eGFR, histological total activity score and fatigue [50]. A twin phase 3 trial in adults and adolescents with primary Ig-MPGN is scheduled to start soon (NCT05755386, Fig. 3) [51].

Drugs that unselectively target C3 activation are also being tested in C3G and Ig-MPGN. Pegcetacoplan is a synthetic cyclic peptide conjugated to a polyethylene glycol polymer that binds to C3 and inhibits C3 activation from all three pathways. Pegcetacoplan also binds to C3b and prevents the activity of the C3 and C5 convertases of the alternative pathway (Fig. 1). The safety and efficacy of pegcetacoplan have been investigated in a phase 2 open-label study (DISCOVERY, NCT03453619) that recruited 21 patients with different glomerulopathies. Results from the seven C3G patients who completed 48 week treatment showed a six-fold increase in serum C3 and a 57.3% decrease in plasma sC5b-9 levels, indicating that pegcetacoplan was able to counteract complement hyperactivity at both C3 and C5 level (Fig. 3). During treatment, there was a 50.9% reduction in proteinuria and an increase in serum albumin [52]. A phase 2 open-label randomized study to evaluate the efficacy of twice-weekly subcutaneous doses of pegcetacoplan in treatment of post-transplant recurrence of C3G or Ig-MPGN [NOBLE (Study Assessing the Safety and Efficacy of Pegcetacoplan in Post-Transplant Recurrence of C3G or IC-MPGN), NCT04572854] [53] has recently closed enrolment (https://noblevalianthcp.com/). A phase 3, placebo-controlled, double-blinded study in adult and adolescent patients with C3G or primary Ig-MPGN (with or without previous renal transplant) is ongoing [NCT05067127, VALIANT (Phase III Study Assessing the Efficacy and Safety of Pegcetacoplan in Patients With C3 Glomerulopathy or Immune-Complex Membranoproliferative Glomerulonephritis), Fig. 3]. The primary endpoint is the proportion of subjects with a reduction from baseline in uPCR of at least 50% at Week 26 [54].

There is also an increasing interest in the exploration of RNA modifying therapies, which could target the complement system. This approach has potential to overcome the challenge of frequent dosing, and to achieve massive inhibition of the target. Among them, ARO-C3 is an investigational RNA interference (RNAi) treatment designed to silence liver-produced C3. A phase 1/2a dose-escalating study (NCT05083364) is currently recruiting participants to evaluate the safety, tolerability, pharmacokinetics and/or pharmacodynamics of ARO-C3. In phase 1, healthy subjects will receive either one or two subcutaneous injections of ARO-C3 or placebo. In phase 2, adult patients with C3G or IgAN will receive three open-label injections of ARO-C3. Dose levels will be determined based on phase 1 data. Interim results of phase 1 are promising, and showed 88% mean reduction of serum C3 and 91% mean reduction in AH50 at the highest dose tested with good safety and tolerability (https://www.bloomberg.com/press-releases/2023-02-28/arrowhead-announces-interim-results-from-ongoing-phase-1-2-study-of-aro-c3-for-treatment-of-complement-mediated-diseases). IONIS-FB-LRX, an antisense oligonucleotide targeting factor B mRNA in the liver is being tested in an open-label phase 2 study in patients with IgA nephropathy (NCT04014335) [41].

Finally, phase 2 studies have been planned in patients with C3G and other nephropathies with other complement inhibitors, including the bifunctional KP104, a monoclonal anti-C5 antibody linked to the FH regulatory domain, which simultaneously inhibits the common terminal pathway and the alternative pathway C3 convertase (NCT05517980), and the anti-Bb humanized monoclonal antibody NM8074 (NCT05647811). New complement targeting agents are moving to clinic, expanding the therapeutic potential for C3G and Ig-MPGN.

Box 2: Strategies for how to personalize treatments. • Patients with nephrotic syndrome or progressive deterioration of renal function despite renoprotective therapy and with no genetic complement abnormalities:MMF and oral prednisone can be attempted, but the optimal duration of treatment, and long-term benefits are uncertain.

• Patients with acute crescentic lesion, and patients with mixed TMA/C3G lesions: anti-C5 treatment may be of benefit.

The cluster approach [5]:

• Cluster 1 and 2 patients, with intense alternative pathway activation in the circulation until the terminal pathway: might respond to anti-FB or anti-FD inhibition but also may benefit from the anti-inflammatory action of C5-inhibitors.

Cluster 2 patients that also show evidence of classical pathway activation: may need a more broad inhibition of C3 via all complement pathways.

Cluster 3 patients who suffer from predominant alternative pathway C3 convertase dysregulation: likely to respond to FB or FD inhibitors rather than to C5 inhibition.

Cluster 4 patients: emerging complement inhibitors that target complement activation on cell surfaces such as FH and CR1 derivatives, might be helpful for blocking solid-phase restricted alternative pathway activation in these patients, while avoiding the potential side effect of broad complement inhibition.

## SUMMARY

Diagnosis of C3G and Ig-MPGN is made by kidney biopsy and should be followed by a comprehensive evaluation of biochemical, genetic and acquired abnormalities of the alternative complement pathway. A universally effective treatment is lacking for primary forms and there is a high rate of progression to ESRD, and disease recurrence in the allograft is very frequent.

Therefore, specific therapies are crucially required to improve the natural history of disease. Complement-targeting treatments may provide a breakthrough in managing these diseases and several trials are ongoing or planned. However, when thinking about complement inhibition in C3G and Ig-MPGN, it is important to consider which component of the cascade may be the most appropriate target for each patient. It is also important to verify what dosages to use to optimize treatment efficacy. For example, responses to FD inhibition may greatly vary, depending on the degree of alternative pathway dysregulation and on FD levels.

Ideally, the choice of drug should be tailored to each patient's characteristics and the underlying specific disease pathogenesis. In this regard, the unsupervised clustering approach may be useful to predict the response to anti-complement therapies (see Box 2).

The risks of new anti-complement agents remain to be quantified, and it should be taken into account that drugs that target complement may reduce the patient's defences against bacterial infections and predispose to autoimmunity. Drugs that inhibit C3 or selectively the alternative pathway may theoretically have greater impact on the opsonisation and lysis of microbes than terminal pathway inhibitors. All patients before receiving complement inhibition should get vaccination against meningococcus and other encapsulated bacteria (*Haemophilus influenzae* and pneumococcus) and in selected cases antibiotic prophylaxis for the duration of treatment. Antibiotic prophylaxis is not required by C5a/C5aR inhibitors, which do not affect the formation of C5b-9 or of the upstream components of complement cascade, and therefore do not affect the lysis of microorganisms. However, reactivation of HBV occurred in trials of avacopan, and the Food and Drug Administration recommends that patients with active, serious infections should avoid using this drug (https://www.fda.gov/drugs/news-events-human-drugs/fda-approves-add-drug-adults-rare-form-blood-vessel-inflammation).

## Supplementary Material

gfad182_Supplemental_File

## Data Availability

No new data were generated or analysed in support of this research.
