# Supplementary material for: C3G and Ig-MPGN—treatment standard
Source: Nephrol Dial Transplant. 2023 Aug 21;39(2):202–14. doi: 10.1093/ndt/gfad182 (PMC10828209; doi:10.1093/ndt/gfad182)
Supplement: gfad182_Supplemental_File [file gfad182_supplemental_file.docx]

**NDT-00718-2023 R1**

**Supplementary references**

S1. Jones G, Juszczak M, Kingdon E, Harber M, Sweny P, Burns A. Treatment of idiopathic membranoproliferative glomerulonephritis with mycophenolate mofetil and steroids. Nephrol Dial Transplant. 2004 19:3160-4.

S2. Yuan M, Zou J, Zhang X, Liu H, Teng J, Zhong Y, Ding X. Combination therapy with mycophenolate mofetil and prednisone in steroid-resistant idiopathic membranoproliferative glomerulonephritis. Clin Nephrol. 2010;73:354-9.

S3. Häffner K, Michelfelder S, Pohl M. Successful therapy of C3Nef-positive C3 glomerulopathy with plasma therapy and immunosuppression. Pediatr Nephrol. 2015;30:1951-9.

S4. Caliskan Y, Torun ES, Tiryaki TO, et al. Immunosuppressive Treatment in C3 Glomerulopathy: Is it Really Effective? Am J Nephrol. 2017;46:96-107.

S5. Bharati J, Tiewsoh K, Kumar A, et al. Usefulness of mycophenolate mofetil in Indian patients with C3 glomerulopathy. Clin Kidney J. 2018;12:483-487.

S6. Avasare RS, Canetta PA, Bomback AS, et al. Mycophenolate Mofetil in Combination with Steroids for Treatment of C3 Glomerulopathy: A Case Series. Clin J Am Soc Nephrol. 2018;13: 406-413.

S7. Yeter HH, Sütiçen E, Korucu B, et al. Evaluation of Clinical, Laboratory and Treatment Modalities in C3 Glomerulopathy: Single Center Experience. Pril (Makedon Akad Nauk Umet Odd Med Nauki). 2019 ;40:15-23.

S8. Caravaca-Fontán F, Díaz-Encarnación MM, Lucientes L, et al. Mycophenolate Mofetil in C3 Glomerulopathy and Pathogenic Drivers of the Disease. Clin J Am Soc Nephrol. 2020;15:1287-1298.

S9. Ravindran A, Fervenza FC, Smith RJH, De Vriese AS, Sethi S. C3 Glomerulopathy: Ten Years' Experience at Mayo Clinic. Mayo Clin Proc. 2018;93:991-1008.

S10. Ayşe Seda Pınarbaşı, Ismail Dursun, Ibrahim Gokce, et al. Predictors of poor kidney outcome in children with C3 glomerulopathy. Pediatr Nephrol 2021;36:1195-1205.

S11. Vivarelli M, Pasini A, Emma F. Eculizumab for the treatment of dense-deposit disease.

N Engl J Med. 2012;366:1163-5.

S12. Daina E, Noris M, Remuzzi G. Eculizumab in a patient with dense-deposit disease. N Engl J Med. 2012;366:1161-3.

S13. Radhakrishnan S, Lunn A, Kirschfink M, et al. Eculizumab and refractory membranoproliferative glomerulonephritis. N Engl J Med. 2012;366:1165-6.

S14. Bomback AS, Smith RJ, Barile GR, et al. Eculizumab for dense deposit disease and C3 glomerulonephritis. Clin J Am Soc Nephrol. 2012;7:748-56.

S15. Herlitz LC, Bomback AS, Markowitz GS, et al. Pathology after eculizumab in dense deposit disease and C3 GN. J Am Soc Nephrol. 2012;23:1229-37.

S16. McCaughan J A, O'Rourke D M, Courtney A E. Recurrent dense deposit disease after renal transplantation: an emerging role for complementary therapies. Am J Transplant. 2012;12:1046-51.

S17. Levart TK, Ferluga D, Vizjak AMraz J, Kojc N. Severe active C3 glomerulonephritis triggered by immune complexes and inactivated after eculizumab therapy. Diagnostic Pathology. 2016; 11:94.

S18. Kerns E, Rozansky D, Troxell ML. Evolution of immunoglobulin deposition in C3-dominant membranoproliferative glomerulonephritis. Pediatr Nephrol. 2013; 28:2227-2231.

S19. Sevgi Gurkan 1, Billie Fyfe, Lynne Weiss et al. Eculizumab and recurrent C3 glomerulonephritis. Pediatr Nephrol. 2013;28:1975-81.

S20. Payette A, Patey N, Dragon-Durey MA, Frémeaux-Bacchi V, Le Deist F, Lapeyraque AL. A case of C3 glomerulonephritis successfully treated with eculizumab. Pediatr Nephrol. 2015;30:1033-7.

S21. Caroline Rousset-Rouvière , Mathilde Cailliez, Florentine Garaix, Daniele Bruno, Daniel Laurent, Michel Tsimaratos. Rituximab fails where eculizumab restores renal function in C3nef-related DDD. Pediatr Nephrol. 2014;29:1107-11.

S22. Oosterveld MJ, Garrelfs MR, Hoppe B, et al. Eculizumab in Pediatric Dense Deposit Disease. Clin J Am Soc Nephrol. 2015;10:1773-82.

S23. Inman M, Prater G, Fatima H, Wallace E. Eculizumab-induced reversal of dialysis-dependent kidney failure from C3 glomerulonephritis. Clin Kidney J. 2015;8:445-8.

S25. Tran CL, Sethi S, Murray D, et al. Discontinuation of dialysis with eculizumab therapy in a pediatric patient with dense deposit disease. Pediatr Nephrol. 2016;31:683-7.

S25. Le Quintrec M, Lionet A, Kandel C, et al. Eculizumab for treatment of rapidly progressive C3 glomerulopathy. Am J Kidney Dis. 2015;65:484-9.

S26. Célia Lebreton, Justine Bacchetta, Frédérique Dijoud, et al. C3 glomerulopathy and eculizumab: a report on four paediatric cases. Pediatr Nephrol. 2017 ;32:1023-1028.

S27. Welte T, Arnold F, Kappes J, et al. Treating C3 glomerulopathy with eculizumab. BMC Nephrol. 2018;19:7.

S28. Garg N, Zhang Y, Nicholson-Weller A, et al. C3 glomerulonephritis secondary to mutations in factors H and I: rapid recurrence in deceased donor kidney transplant effectively treated with eculizumab. Nephrol Dial Transplant. 2018;33:2260-2265.

S29. Ozkaya O, Nalcacioglu H, Tekcan D, et al. Eculizumab therapy in a patient with dense-deposit disease associated with partial lipodystropy. Pediatr Nephrol. 2014;29:1283-7.

S30. Sánchez-Moreno A, De la Cerda F, Cabrera R, et al. Eculizumab in dense-deposit disease after renal transplantation. Pediatr Nephrol. 2014;29:2055-9.

S31. Besbas N, Gulhan B, Gucer S, Korkmaz E, Ozaltin F. A novel CFHR5 mutation associated with C3 glomerulonephritis in a Turkish girl. J Nephrol. 2014;27:457-60.

S32. Carrara C, Podestà MA, Abbate M, et al. Morphofunctional Effects of C5 Convertase Blockade in Immune Complex-Mediated Membranoproliferative Glomerulonephritis: Report of Two Cases with Evidence of Terminal Complement Activation. Nephron. 2020;144:195-203.

S33. Giuseppina Spartà, Ariana Gaspert, Thomas J Neuhaus, et al. Membranoproliferative glomerulonephritis and C3 glomerulopathy in children: change in treatment modality? A report of a case series. Clin Kidney J. 201;11:479-490.

S34. Chanchlani R, Thorner P, Radhakrishnan S, et al. Long-term Eculizumab Therapy in a Child With Refractory Immune Complex-Mediated Membranoproliferative Glomerulonephritis. Kidney Int Rep. 2017;3:482-485.

S35. Holle J, Berenberg-Goßler L, Wu K, et al. Outcome of membranoproliferative glomerulonephritis and C3-glomerulopathy in children and adolescents. Pediatr Nephrol. 2018;33:2289-2298.

S36. Sahin H, Gok Oguz E, Akoglu H, et al. Successful Treatment of Posttransplant Recurrent Complement C3 Glomerulopathy with Eculizumab. Iran J Kidney Dis. 2018;12:315-318.

S37. Kojc N, Bahovec A, Levart TK. C3 glomerulopathy in children: Is there still a place for anti-cellular immunosuppression? Nephrology (Carlton). 2019;24:188-194.

S38. Renu Regunathan-Shenk, Rupali S Avasare, Wooin Ahn, et al. Kidney Transplantation in C3 Glomerulopathy: A Case Series. Am J Kidney Dis. 2019;73:316-323.

S39. Yazılıtaş F, Kargın Çakıcı E, et al. C3 glomerulopathy: experience of a pediatric nephrology center. Acta Clin Belg. 2021;76:253-257.

S40. Katsuaki Kasahara, Yoshimitsu Gotoh, Hisakazu Majima, Asami Takeda, Masashi Mizuno.

Eculizumab for pediatric dense deposit disease: A case report and literature review. Clin Nephrol Case Stud. 2020;8:96-102.

S41. Blatt NB, Kumar T, Wickman LT, Kanaan HD, Chang A, Zhang PL. Myeloperoxidase immunohistochemical staining can identify glomerular endothelial cell injury in dense deposit disease. Pediatr Nephrol. 2021;36:4003-4007.

S42. Muhammad Saad Naseer, Ayush Singh, Neeraj Singh. Repository Corticotropin in Treating de novo C3 Glomerulonephritis after Transplantation. Glomerular Dis. 2021;2:100-105.

S43. Elia Balestra, Egidio Barbi, Viola Ceconi. Pioglitazone, a PPAR-y agonist, as one of the new therapeutic candidates for C3 glomerulopathy Pediatr Nephrol. 2023 Jul 26 2023 Online ahead of print.
